# Supplementary material for: Impact of medication therapy management service on selected clinical and humanistic outcomes in the ambulatory diabetes patients of Tikur Anbessa Specialist Hospital, Addis Ababa, Ethiopia
Source: PLoS One. 2021 Jun 2;16(6):e0251709. doi: 10.1371/journal.pone.0251709 (PMC8171943; doi:10.1371/journal.pone.0251709)
Supplement: S1 File — (DOCX) [file pone.0251709.s001.docx]

**Supporting information**

**Data collection tool.**

Appendixes I

**Appendix A (English version)**

Pharmacy Contact Information Here

Pharmacist: ______________________

1. **Patient information**

Age (year):_____ Sex: ____

| Marital status: | Single | | Married | Divorced Widowed |
| --- | --- | --- | --- | --- |
| Educational status | Unable to write & read | | Informal education  primary school | Secondary school  College diploma and above |
| Residence(current): __________________________________ | | | | |
| Occupation | Employed Unemployed Private Student  Other(specify)___________________ | | | |
| How do get your medication? | | Buying Free Company Third Party Coverage 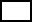 | | |

Treating physician _____________________________________________

Caregiver (if applicable) ________________________ phone no._________________________

Pharmacist completing review _________________________________

1. **Health Information and Lifestyle Factors**

| **Inquiry** | **Yes/No** | **Details/ comments** |
| --- | --- | --- |
| 1. Allergies | Y N | Reaction |
| 1. Smoker | Y N | Cigarettes/day: |
| 1. Alcohol Consumption | Y N | Drinks/week: |
| 1. Caffeine Intake | Y N | Cups/day: |
| 1. Grapefruit (Juice) Consumption | Y N | Comments: |
| 1. Restricted Diet | Y N | Specify : |
| 1. Physically Active | Y N | Type of activity: Hours/week |
| 1. Recreational/Other Drug Use | Y N | Specify: |
| 1. BMI(kg/m^2^) | Normal  Overweight  Underweight  Obese  Morbid obese | Height(m):_____Weight(kg)____ |
| 1. WC(cm) | Normal  Increased |  |
| 1. Do you have person assist you in medication use | Y N |  |
| 1. Aids, Alerts, Devices, etc. | Others |  |

Past medical history (relevant illnesses, hospitalizations, surgical procedures, injuries, pregnancies).

Past medication(s)______________________________________________________________

Family History (FH): ____________________________________________________________

Functional History (if relevant- i.e. geriatrics, stroke patient, homeless, etc.): ______________________________________________________________________________

Physical Examination (PE)/**vital** signs:

| **Parameters** | **Date(dd/mm/yy** |  |  |  |  |  |  |  |  |  |  |
| --- | --- | --- | --- | --- | --- | --- | --- | --- | --- | --- | --- |
| BP |  |  | | |  | | |  |  |  |  |
| PR |  |  | | |  | | |  |  |  |  |
| RR |  |  | | |  | | |  |  |  |  |
| T^0^ |  |  | | |  | | |  |  |  |  |
| Others: |  |  | | |  | | |  |  |  |  |
|  | | | | | | | | | | | |

Relevant **laboratory** series results (Lab Findings of at least for three consecutive results).

| **Parameters** | Date(dd/mm/yy |  |  |  |  |  |  |  |  |  |  |
| --- | --- | --- | --- | --- | --- | --- | --- | --- | --- | --- | --- |
| HbA1c (%): |  |  | | |  | | |  | | |  |
| FBS(mg/dL): |  |  | | |  | | |  | | |  |
| RBS(mg/dL): |  |  | | |  | | |  | | |  |
| Lipid profiles | LDL: mg/dl |  | | |  | | |  | | |  |
|  | TG: mg/dl |  | | |  | | |  | | |  |
|  | HDL: mg/dl |  | | |  | | |  | | |  |
|  | Total C |  | | |  | | |  | | |  |
| OFTs | ALT/SGOPT |  | | |  | | |  | | |  |
|  | AST/SGPT |  | | |  | | |  | | |  |
|  | ALP |  | | |  | | |  | | |  |
|  | GFR |  | | |  | | |  | | |  |
|  | SrCr |  | | |  | | |  | | |  |
| Others |  |  | | |  | | |  | | |  |
|  |  |  | | |  | | |  | | |  |

**Current Medical Conditions** *(List medicalconditions in numbered spaces with relevant information/parameters)*

| 1. | 2. | 3. | 4. |
| --- | --- | --- | --- |
| 5. | 6. | 7. | 8. |
| **Head to toe Assessment regarding othercomplaints/concerns/bothersomesymptoms:**  Complaints/Concerns:  Bothersome symptoms:  Do any ever require self-treatment? | | | |

Medications (Prescription, Non-Prescription, Herbal Products)

| Medication  Name, strength | How taken  Dose, route, frequency, time of day, special instruction | Purpose for use | Starting date | Stopped date | Who stopped it? Reason for stopping | Issues identified | | Additional comments |
| --- | --- | --- | --- | --- | --- | --- | --- | --- |
|  |  |  |  |  |  | Yes:  proceed to DTPs identified | No:  verify to continue as per |  |
|  |  |  |  |  |  |  | |  |
|  |  |  |  |  |  |  | |  |
|  |  |  |  |  |  |  | |  |

**Drug Therapy Problems Identified and Addressed by MTM Pharmacists**

| DTP type | Categories of DTP | Drug therapy problem cause |
| --- | --- | --- |
| Indication | 1. Unnecessary drug therapy | - Duplicate therapy - No medical indication at this time - Nondrug therapy more appropriate - Addiction/recreational drug use - Treating avoidable adverse reaction |
|  | 2. Needs additional drug therapy | - Preventive therapy - Untreated condition - Synergistic therapy |
| Effectiveness | 3. Ineffective drug | - More effective drug available - Condition refractory to drug - Dosage form inappropriate - Contraindication present - Drug not indicated for condition |
|  | 4. Dosage too low | - Ineffective dose - Needs additional monitoring - Frequency inappropriate - Incorrect administration - Drug interaction - Incorrect storage - Duration inappropriate |
| Safety | 5. Adverse drug reaction | - Undesirable effect - Unsafe drug for the patient - Drug interaction - Incorrect administration - Allergic reaction - Dosage increase/decrease too fast |
|  | 6. Dosage too high | - Dose too high - Needs additional monitoring - Frequency too short - Duration too long - Drug interaction |

**Drug therapy problems identified**

No drug therapy problems were identified

**Priority Number Drug Therapy Problem (DTP)**

________________________________________________________________________________________________________________________________________

For those drug therapy problems above which can be corrected with *immediate action* and *no further research or consultation*, document your plan below:

| DTP # | Proposed solution | Discussed with patient | Follow-up plan |
| --- | --- | --- | --- |
|  |  |  |  |
|  |  |  |  |
|  |  |  |  |

For those drug therapy problems requiring *further research, contact with other health care providers* and *care plan development*, utilize the **Pharmacy Care Plan** worksheet.

**Pharmacy Care Plan**

**Data:** Subjective information provided by the patient and/or objective data that you have collected.

**Assessment:** State the drug therapy problem.

**Plan:** For each alternative, consider *treatment efficacy, safety, drug interactions, adherence, cost, drug cover age* and *non-pharmacological interventions*.

**Alternative#1:**

**Alternative#2:**

**Monitoring:**

**Planned date of follow-up:**____________________________

Pharmacist signature__________ Date of Review___________

**Patient action plan**

Date of comprehensive medication review:_________________________________

As a result of comprehensive medication review, I will do the following:

|  |
| --- |
|  |
|  |

**Patient follow record**

| Date follow up | Reason for follow-up | Results | Pharmacist comments &plan |
| --- | --- | --- | --- |
|  |  | Any new concerns? | Intervention complete?  Yes No |
|  |  | Any new concerns? | Intervention complete?  Yes No |
|  |  | Any new concerns? | Intervention complete?  Yes No |

**Health Care Practitioner Communication Form**


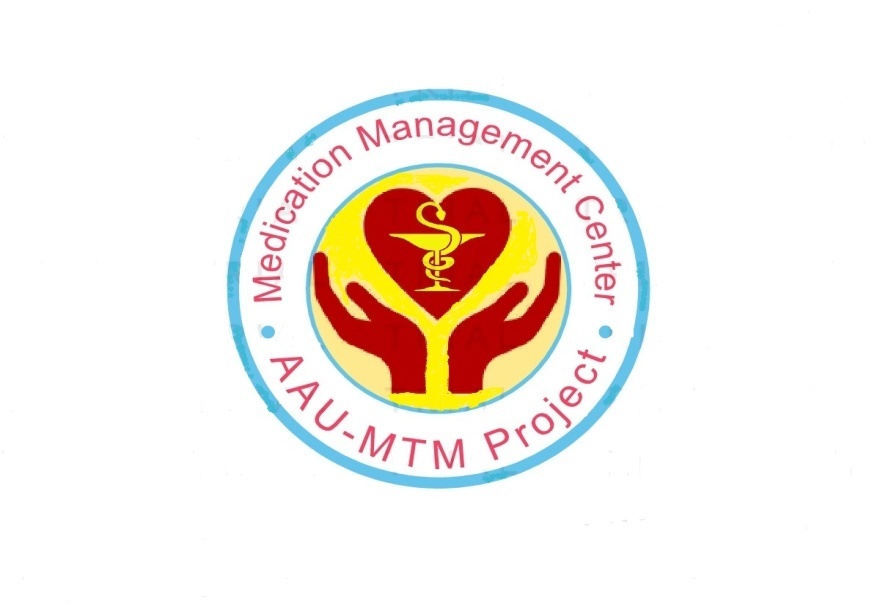


**Date:_______________________**

| Healthcare Practitioner | Re:(Patient’s Name) |
| --- | --- |
| Address | Address |
| Phone# | Age Phone # |

Dear Dr.___________________

Your patient had a Comprehensive Medication Review completed on--------------. Listed below are my assessment(s) and recommendation(s).Please provide a response below (if indicated) at your earliest opportunity.Should you like to discuss any ofthe information contained don’thesitate to contactme.

| Drug Therapy Problem | PharmacistRecommendation | | Make Changes as Recommended | PrescribeComments/Revisions |
| --- | --- | --- | --- | --- |
|  | Information Only | Action Required | Yes No |  |
|  |  |  |  |  |
|  | Information Only | Action Required | Yes No |  |
|  |  |  |  |  |
| PharmacistName: | | | Prescriber Signature:____ Date _____ | |

Compliance information

Morisky 8-Item Medication Adherence Questionnaire

Scores: >2 = low adherence; 1 or 2 = medium adherence; 0 = high adherence

| Questions | Patient Answer Score(Yes=1;No=0) |
| --- | --- |
| Do you sometimes forget to take your medicine? |  |
| People sometimes miss taking their medicines for reasons other than forgetting. Thinking over the past 2 weeks, were there any days when you did not take your medicine? |  |
| Have you ever cut back or stopped taking your medicine without telling your doctor because you felt worse when you took it? |  |
| When you travel or leave home, do you sometimes forget to bring along your medicine? |  |
| Did you take all your medicines yesterday? |  |
| When you feel like your symptoms are under control, do you sometimes stop taking your medicine? |  |
| Taking medicine every day is a real inconvenience for some people. Do you ever feel hassled about sticking to your treatment plan? |  |
| How often do you have difficulty remembering to take all your medicine?   1. Never/rarely 2. Once in a while 3. Sometimes 4. Usually 5. All the time | A = 0;  B-E = 1 |
|  | Total score |

Possible reasons/determinants for poor medication Adherence:

| Fear of medication adverse events | Disbelief in drug effectiveness |
| --- | --- |
| Inadequate instruction | Patient prefers not to take |
| Inadequate availability of medication | Difficulty of administration |
| Simply Forgetfulness | Regimen complexity |
| Feeling better or worse | Cost of medication too expensive |
| Due to work load/busy | Others …......................... |

**Part IV. Assessment of adverse drug reaction (undesirable effect)**Have you experienced any undesirable, unusual adverse drug events /allergic reaction to
the prescribed medicines? Yes No:
If yes would you describe the manifestation of the events ---------------------------------

| Hypoglycemia |  |  | Epigastric pain |  |  | Head ache |  | |  | |  | | Diarrhea/constipation | | |  | Weight gain |  | |  | |
| --- | --- | --- | --- | --- | --- | --- | --- | --- | --- | --- | --- | --- | --- | --- | --- | --- | --- | --- | --- | --- | --- |
|  |  |  |  |  |  |  |  | |  |  |  |  |  |  |  |  |  |  | |  |  |
| Irritability | |  | Blurred vision |  |  | Depression | |  | |  |  | | Weakness |  |  | | Hypersomnia | |  | |  |
|  |  |  |  |  |  |  |  |  | |  |  |  |  |  |  |  |  |  |  | |  |
| Forgetfulness |  |  | Skin rash |  | | Confusion | | | | |  |  | Others please specify ------------------- | | | | | | | | |

Satisfaction Assessment Questionnaire

Available at ;

https://eprovide.mapi-trust.org/content/download/28321/283388/version/1/file/ SATMED-Q_AU1.0_eng-GB_ReviewCopy.pdf

Annex: Questionnaire, Amharic Version (የአማርኛ መጠይቅ ቅፅ)

Card number____________________ Code number----------- date------------

ቅፅ 3: ቃለ-መጠይቅ ከታካሚው

| ክፍል 1፡ ስለታካሚው አጠቃላይ መገለጫዎች**:** | | | | | | |
| --- | --- | --- | --- | --- | --- | --- |
| 1. ዕድሜ (በአመት):_____ | |  | | |  |  |
| 2.ጾታ | | ወንድ | | | ሴት |  |
| 3. የጋብቻ ሁኔታ: | | ያላገባ*/*ያላገባች | | | ያገባ*/*ያገባች የፈታ/ች | ባል የሞተባት */*ሚስት የሞተችበት |
| 4.የትምህርት ደረጃ | | ያልተማረ*/*ች 1-8ኛ ክፍል | | | 9-10ኛክፍል 10-12ኛ ክፍል | ኮሌጅ ድፕሎማና በላይ |
| 5.የመኖርያ ቦታ | | አዲስ አበባ ከአዲስ አበባ ዉጪ | | | | |
| 6. የስራ ሁኔታ | | ተቀጣሪ ስራ አጥ የግል ስራ*/*ነጋዴ ተማሪ ሌላ*/ሌሎ*ች *(*ይገለፅ)_ | | | | |
| 7.ወርሓዊ ጠቅላለ የቤተሰብ ገቢ(በብር) | | | | <1500 1500-3000 3000-5000 >5000 | | |
| 8. ማህበራዊ ልማድ ሁኔታ | 8.1. ሲጋራ ያጨሳሉ? | | አዎ አለጨስም | | | <1እሽግ በሰምንት  >1 እሽግ በሰምንት  ድሮ አጨስ ነበር አሁን አቁሜያለሁ |
|  | 8.2.መጠጥ *(*የአልኮል*)*? | | አዎ አልጠጣም | | | በቀን ስንት? _________ |
|  | ጫት ይቅማሉ? | | አዎ አልቅምም | | | ድሮ እቅም ነበር አሁን አቁሜያለሁ |
| 9.የአከል ብቃት እንቅስቃሴ | በእግር መጓዝ | አዎ | አለደርግም | | | ካደረጉ ለምን ያህል ጊዜ?  በቀንከ30ደቂቃበታች በቀን ከ30ደቂቃ በላይ |
|  | ስፖርት | አዎ | አልሰራም | | | ከሰሩ ለምን ያህል ጊዜ?  በየቀኑ 1-3ቀን በሳምንት  4-6ቀን በሳምንት |
| ክፍል 2፡ ስለህክምና አጠቃላይ መገለጫዎች | | | | | | |
| 1.የስኳር ህመምዎት ተመርምረው ካወቁ ምን ያህል ዓመት ሆኖታል? ____________ | | | | | | |
| 2.ለዚህ ህመምዎ መዴኃኒት መውሰዴ ከጀመሩ ምን ያህሌ ዓመት ነው? _________ | | | | | | |
| 3.ህመሙን ካወቁ በኋላ ሆስፒታል ገብቶ ያዉቃሉ? | | አዎ | የለም | | አዎ ከሉ በምን ምክንያት; በስኳር መብዛት በስኳር መነስ ሌላ ከለ እባክዎን ይግለጹት__ | |
| 4. ለዚህ ህመምዎ የሚወስዱት መዴሃኒት | | አንድ የሚዋጥ________________________________________  አንድ የሚዋጥ + ኢንሱሊን________________________________  2 የሚዋጥ ብቻ________________________________________  2 የሚዋጥ + ኢንሱሊን___________________________________  3 የሚዋጥ ብቻ________________________________________  ኢንሱሊን ብቻ፣ ጥቀስ__________________________________ | | | | |
| 5. ለስኳር ህመምዎ ከሚወስዱት ሌላ ተጨማሪ ቋሚ መዴኃኒቶች ወይም *(*የባህል ወይም ያላሓኪም ትእዛዝ የሚወሰዱት መዴኃኒቶችን*)* አለ? | | የለም አዎ, ካላ እባክዎን ይግለጹት ______________________ እና ለምን አገልግሎት ____________________________________________ | | | | |
| 6. ተጨማሪ ወይም ሌላ ተያያዥ ህመም አለብዎት? | | የለም አለ , ካለ እበክዎ ይግለጹ ______________________________ | | | | |
| 7. በህመሙ ምክንያት የመጣ ችግር | | የለም አለ, ካለ እበክዎ ይግለጹ ____________________ | | | | |
| 8. የመድህኒት ከሰውነት ጋር አለመስማማት) | | የልም አለ, ካለ መድህኒቱ ይገለጽ _______________________ | | | | |
| 9. መድሃኒት የሚያገኙት በምን መልኩ ነው? | | በግዢ በነጻ ከድርጅት | | | | |

ክፍል**2:** ሞሪስኪ**”** መድኃኒትን በታዘዘው መሰረት በአግባቡ ስለመውሰድ**”** መለኪያ**- 8**

| ተ.ቁ | ጥያቄዎች | አዎ | አይደለም |
| --- | --- | --- | --- |
| 1 | አንዳንድ ጊዜ መድኃኒትዎን ረስተው ሳይወሰዱ ቀርተው ያውቃለ? | 1 | 0 |
| 2 | ሰዎች አንዳንድ ጊዜ ከመርሳት በተጨማሪ ባሉት የተለያዩ ምክንያቶች መድኃኒታቸውን ሳይወስዱ ይቀራሉ፡፡ባለፉት ሁለት ሳምንታት፣ መድኃኒትዎን ሳይወስዱ የቀሩበት ቀናቶች ነበሩ? | 1 | 0 |
| 3 | መድኃኒትዎን እየወሰዱ ህመምዎ ባለመቆሙ ሐኪምዎን ሳያማከሩ መድኃኒትዎን አቋርጠው ያውቃሉ? | 1 | 0 |
| 4 | በጉዞ ወይም በሌላ ምክንያት ከቤትዎ እርቀው ሲጓዙ አንዳንድ ጊዜ መድኃኒትዎን ረስተውት ሳይወስዱት ያውቃለ? | 1 | 0 |
| 5 | በትላንትናው ዕለት ሁሉንም መድኃኒትዎን ውጠዋል? | 0 | 1 |
| 6 | ህመምዎ ጋብ ሲልሎት (የህመምዎ ስሜቶች ሲጠፈ) አንደንዴ ጊዜ መድኃኒትዎን አቋርጠው ያውቃሉ? | 1 | 0 |
| 7 | በየቀኑ መድኃኒት መዋጥ፣ ለአንዳንድ ሰዎች አይመችም፡፡ እርስዎ በየቀኑ እንድሁም አንዴም ሰዓት ሳያዛንፉ መድኃኒትዎን መዋጥ የመሰለቸት ስሜት ተሰምቶት ያውቃሉ? | 1 | 0 |
| 8 | መድኃኒትዎን አስታውሰው ለመዋጥ ምን ያክል ይቸገራሉ?  ጭራሽ አይቸግረኝም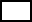በጣም አልፎ አልፎ ከስንት አንድ ጊዜ ይቸግረኛል  አንዳንድ ጊዜ ይቸግረኛልአብዛኛው ጊዜ ይቸግረኛል ሁሌጊዜ ይቸግረኛል |  |  |

2.1 መድሃኒቱን በአግባቡ ካልወሰዱ እባክዎ ምክንያት ይግለጹ (ከአንድ በላይ መልስ መምረጥ ይቻላል) .....

| የጎኒዮሽ ጉዲትን (ሳይዴኢፌክት) በመፍራት | ያድነኛል ብዬ ስለማላስብ |
| --- | --- |
| ስለመድሃኒቱን አወሳሰድ በቂ መረጃ ስለሌለኝ | መድሃኒቱን ስወስድ ህመሜ ስለሚባባስበኝ |
| መድሃኒቱንማግኘትስላልቻልኩ | መድሃኒቱን ስውጠው ስለሚያስቸገረኝ |
| ስለምረሳው | የምወስዳቸው መድሃኒቶች ብዙና ግራ የሚያጋቡ ስለሆኑ |
| ተሽሎኛል ብዬ ስላሰብኩ | መድሃኒቱ ውድ ስለሆነ |
| ስራ ስለምበዛብኝ | ሌላም ካለ ይግለፁት………......................... |

ክፍል3 ከመድኃኒትዎ የጎኒዮሽ ጉዲት (ሳይዴኢፋክት) ግምገማን በተመለከተ

3.1 በአሁን ሰዓት ወይም መድሃኒትዎን መውሰድ ሲጀምሩ ከመድሃኒቱ ጋር የተያያዙ ያልተለመዱ ሁኔታዎች/ የጎኒዮሽ ጉዲት አጋጥሞዎት ያውቃል። አዎ አላጋጠመኝም

3.2 መሌስዎ አዎን ከሆነ የመድሃኒቱን ስሙና የነበረው ሁኔታ ይግለጹ (ከአንድ በላይ መልስ መምረጥ ይቻላል)

| የስኳርመቀነስ |  |  | የጨጓራ ህመም |  |  | የራስ ምታት |  | |  | |  | ተቅማጥ/ድርቀት | | | |  | የክብደት መጨመር |  | |  | |
| --- | --- | --- | --- | --- | --- | --- | --- | --- | --- | --- | --- | --- | --- | --- | --- | --- | --- | --- | --- | --- | --- |
|  |  |  |  |  |  |  |  | |  |  |  |  |  |  |  |  |  |  | |  |  |
| ግራ መጋባት | |  | የአይን ብዥታ |  |  | ድብርት | |  | |  |  | የዴካም ስሜት | |  |  | | ከመጠን ያለፈ እንቅልፍ | |  | |  |
|  |  |  |  |  |  |  |  |  | |  |  |  |  |  |  |  |  |  |  | |  |
| መርሳት |  |  | የቆዳ ላይ ሽፊታና ማሳከክ |  | | ራስንማዞር (ብዥታ) | | | | |  |  | ሌላም ካለ ይግለፁት------------------- | | | | | | | | |

**SATMED-Q** የታካሚዎች ስለሚወስዱት መድሐኒት**/**ህክምና ያላቸውን እርካታ መመዘኛ መጠይቅ

**መመሪያ**፡ለእያንዳንዳቸው ጥያቄ የእርስዎን ስሜት ይበልጥ ይገልጥልኛ ልያሉትን ከተሰጡት የእረካታ መመዘኛ አማራጮች መካከል አንዷን ይምረጡ፡፡ ለጥያቄዎቹ ትክክለኛ ወይም የተሳሳተ ምለሽ የላቸዉም፡፡ ለሚሰጡት ምላሽ እርግጠኛ ካልሆኑ ይበልጥ ይስማማኛል ብለዉ ያሰቡትን ሀሳብ የያዘለወትን ምርጫ ይምረጡ፡፡

በጭራሽ = 0, በትንሹ = 1, በመጠኑ = 2, በጣም = 3 ,እጅግ በጣም = 4

|  |  | | በጭራሽ | በትንሹ | በመጠኑ | በጣም | እ.በጣም |
| --- | --- | --- | --- | --- | --- | --- | --- |
|  | ይህ ክፍል ስለመድሃኒቱ የጎዮሸ ጉዳት ይመለከታል፡፡ | | | | |  |  |
| 1 | የመድሃኒቱ የጎንዮሽ ጉዳት በአካላዊ እንቅስቃሴ ላይ ተጽእኖ አሳድሯል | | 0 | 1 | 2 | 3 | 4 |
| 2 | የመድሃኒቱ የጎንዮሽ ጉዳት በእረፍት እና በትርፍ ጊዜዬ ላይ ተጽእኖ አሳድሯል | | 0 | 1 | 2 | 3 | 4 |
| 3 | የመድሃኒቱ የጎንዮሽ ጉዳት በጠቅለላው የእለት ተእለት እንቅስቃሴዬ ላይ ተጽእኖ አሳድሯል | | 0 | 1 | 2 | 3 | 4 |
|  | ይህ ክፍል ስለመድሐኒቱ ዉጤታማነት ማለትም በሽታዉን ወይም የበሽታዉን ምልክት ስለማከሙ ይገልፃል፡፡ | | | | |  |  |
| 4 | የምወስዳዉ መድሐኒት የበሽታዬን ምልክቶች አጥፍቶአቸዋል፡፡ | | 0 | 1 | 2 | 3 | 4 |
| 5 | መድሐኒቱ ከወሰድኩ በኋላ ቶሎ ለውጥ ስላማይበት ረክቻለሁ፡፡ | | 0 | 1 | 2 | 3 | 4 |
| 6 | በፊት ከነበረኝ የጤና ሁኔታ ይልቅ ህክምና ከጀመረኩ በኋለ ጥሩ ስሜት እየተሰማኝ ነዉ፡፡ | | 0 | 1 | 2 | 3 | 4 |
|  | ይህ ክፍል ስለመድሐኒቱ ምቹነት ወይም ለአወሳሰድ ቀላል ስለመሆን አለመሆኑ ይመለከታል፡፡ | | | | |  |  |
| 7 | መድሐኒቶቼን በቀላሉ መወሰድ የሚችል እንዳሆነ አዉቂያለሁ፡፡ | | 0 | 1 | 2 | 3 | 4 |
| 8 | መድሐኒቶቼ ባለበት ሁኔታ (በጣዕማቸዉ፣ በመጠናቸዉና በመሳሰሉት) በቀላሉ መዉሰድ እንዳምችል አዉቂያለሁ፡፡ | | 0 | 1 | 2 | 3 | 4 |
| 9 | መድሐኒቶቼን የምወስድበት የጊዜ ሰሌዳ ተመችቶኛል፡፡ | | 0 | 1 | 2 | 3 | 4 |
|  | ይህ ክፍል መድሐኒቱ በዕለት ከዕለት ኑሮዎት ለይስ ለሚኖረዉ ሚና ይመለከታል፡፡ | | | | |  |  |
| 10 | ዕድሜ ለምወስዳዉ መድሐኒት በትርፍ ጊዜዬ የምሰራቸዉን ስራዎች ማከናዎን ቀላል ሆኖልኛል ነዉ፡፡ | | 0 | 1 | 2 | 3 | 4 |
| 11 | ዕድሜ ለመዴሐኒቴ የግል ንፅህናዬን ለመጠበቅ ቀላል ሆኖልኛል ነዉ፡፡ | | 0 | 1 | 2 | 3 | 4 |
| 12 | ዕድሜ ለመድሐኒቴ የእልለት ከእለት እንቅስቃሴዬን ለማከናወን ቀላል ሆኖልኛል ነዉ፡፡ | | 0 | 1 | 2 | 3 | 4 |
|  | ይህ ክፍል ስለህክምናዉ ክትትል ይመለከታል፡፡ | | | | |  |  |
| 13 | ሐኪሜ ስለጤናዬ ሁኔታ በጥልቀት አሳዉቆኛል፡፡ | | 0 | 1 | 2 | 3 | 4 |
| 14 | ሐኪሜ ያጋጠመኝ የጤና ችግር በተገቢዉ ሁኔታ እንዴት መታከም እንዲለበት አሳዉቆኛል፡፡ | | 0 | 1 | 2 | 3 | 4 |
|  | በመጨረሻም መጠይቁ ስለመድሐኒቱና ስለታካሚዉ ጤንነት አጠቃላይ ያለዉን ሃሳብምን እንዳሚመስል ይመለከታል፡፡ | | | | | |  |
| 15 | መድሐኒቱን በቀጣይነት ለመዉሰድ አቅጃለሁ |  | 0 | 1 | 2 | 3 | 4 |
| 16 | በሚዳረግሌኝ ህክምና ምቾት ተሰምቶኛል፡፡ |  | 0 | 1 | 2 | 3 | 4 |
| 17 | በአጠቃሊይ በሚደረግሌኝ ህክምና ረክቻለሁ፡፡ |  | 0 | 1 | 2 | 3 | 4 |
|  | ጠቅላላ ውጤት |  |  | | |  |  |
