# Supplementary material for: Impact of medication therapy management service on selected clinical and humanistic outcomes in the ambulatory diabetes patients of Tikur Anbessa Specialist Hospital, Addis Ababa, Ethiopia
Source: PLoS One. 2021 Jun 2;16(6):e0251709. doi: 10.1371/journal.pone.0251709 (PMC8171943; doi:10.1371/journal.pone.0251709)
Supplement: Attachment — Submitted filename: PONE-D-20-27442 REVIEWERS COMMENT.docx [file pone.0251709.s002.docx]

**Title:** Patient outcomes and satisfaction improves following introduction of Medication therapy management service at the ambulatory diabetes clinic of Tikur Anbessa Specialized Hospital, Addis Ababa, Ethiopia

**Manuscript Number:** PONE-D-20-27442

**Article Type:** Research Article

The authors have done well. However, the following corrections should be made to make the work publishable and of good standard:

1. The title, Patient outcomes and satisfaction improves following introduction of Medication therapy management service at the ambulatory diabetes clinic of Tikur Anbessa Specialized Hospital, Addis Ababa, Ethiopia”, is a biased one from the onset. The word improves shows bias. The title should rather be, “**Impact of Medication therapy management service on Patient outcomes and satisfaction in the ambulatory diabetes clinic of Tikur Anbessa Specialized Hospital, Addis Ababa, Ethiopia**”
2. Again, satisfaction is an outcome (humanistic outcome), so it is like a repetition. The authors could modify the title further to ‘sellected clinical and humanistic outcomes’
3. The final title should now be,”**Impact of Medication Therapy Management service on sellected Clinical and Humanistic outcomes in the ambulatory diabetes Patients of a Specialist Hospital in Addis Ababa, Ethiopia**” or ,”**Impact of Medication Therapy Management service on sellected outcomes in the ambulatory diabetes Patients of a Specialist Hospital in Ethiopia**”
4. The fiest sentence at the background section of the abstract is rather long and passive. It should be split into two and the phrase, ‘as they often’ should be exponged. Line 17
5. The third sentence of the background section; (Thus aim of present study was to assess .....) could be rephrased to, (The study assessed .......) Line 19
6. In lines 19- 21, the objective of the study was incomplete eg he impact of MTM on what? Patient outcomes, satisfaction, etc
7. The outcome measures should be clearly delineated because patient outcomes comprises : clinical, economic, and humanistic outcomes. Here, i guess the authors are refaring to clinical outcomes , they should be specific on the them for precision
8. Line 66 should mark the begining of a new paragraph; (It also involves .....) should translate to (Also, it involves .....)
9. The phrase in line 103 (so as double recruitment ....) should be rephrased
10. In line 133, indicate the major modifications made to the questionnaire and reference it
11. How was bias handled/eliminated from the study
12. The authors should clearly state the outcome measures of this study under a sub-title at the methods section of the manuscript
13. In line 106, At the day of appointment, should be (On the day of appointment,)
14. The tables titles were rather to long. The authors should rephrase/restructure the tables titles to be very precise/concise; the name and location of the hospital can be exponged since it is already known from the title of the study.
15. Line 246-251 need additional references to establish the statements made.
16. In line 153, the statement (About 73% had) is undefined. The authord should define this population and be definite.Eg 75% of patients, type 2 diabetic patients, ???
17. Lines 271-272 should be ellaborated with relevant studies/references
18. In line 319 (The intervention period is relatively short) should be corrected
19. Line 298- 303, 319- 324 were poorly discussed and without any reference. The authors should improve on them
20. The study was poorly concluded in lines 325-329.
21. Page number is missing in reference NO 26, line 439. The first year (2011) on that reference should be cross checked.
22. The journal name in reference NO 28, line 446 should be cross checked.
23. Page number is missing in reference NO 36, line 473, and NO 37 line 475 respectively.

Thank you.
