# Supplementary material for: Impact of medication therapy management service on selected clinical and humanistic outcomes in the ambulatory diabetes patients of Tikur Anbessa Specialist Hospital, Addis Ababa, Ethiopia
Source: PLoS One. 2021 Jun 2;16(6):e0251709. doi: 10.1371/journal.pone.0251709 (PMC8171943; doi:10.1371/journal.pone.0251709)
Supplement: Attachment — Submitted filename: Rebuttal letter.docx [file pone.0251709.s003.docx]

**REBUTTAL LETTER**

**Dear Editors,**

Hopefully, all is good with you. As per the request, suggestion, and comment of the editors and reviewers of the manuscript we try to address all the points raised during the revision of the manuscript. The points raised and addressed are described as follow;

**Response to editors**

1. Please ensure that your manuscript meets PLOS ONE's style requirements, including those for file naming.

- *The format was corrected according to the PLOS ONE's style requirements.*
- *Figure, file name, font size, spacing, etc.*

2. In your Methods section, please provide additional information about the participant recruitment method and the demographic details of your participants. Please ensure you have provided sufficient details to replicate the analyses such as: a) a statement as to whether your sample can be considered representative of a larger population, b) a description of how participants were recruited, and c) descriptions of where participants were recruited and where the research took place.

*A sample size(n) of 423 were computed based on single proportion formula assuming DTPs prevalence(p) 50%. A critical value for normal distribution at 95% confidence interval, z-value of 1.96 was used with a margin of error(d) 5% in sample size calculation.*

$$n=\frac{Z^{2} p (1-p)}{d^{2}}=\frac{\left( 1.96 \right)^{2}x (0.5) (0.5)}{{(0.05)}^{2}} = 384.16$$

*Therefore, with adjustment for 10% contingency (for non-response), the total sample size were 423. These number account for more than 7 percent of the population which is a representative sample size considering time and resource limitation.*

*During the three months recruitment periods, patients who had an appointment at the diabetes clinic of TASH were made the sampling frame and the sampling fraction was calculated. The average 125 daily attendees were used for calculation of constant (k). The total sample size (423) was divided by the number of days the clinic provide service within three months (24 days) of the recruitment period to get the estimated sample of participants per day. Based on this calculation about 18 patients were sampled each day. This was made for the purpose of participant distribution throughout the study period for better representativeness. A systematic random sampling technique was used based on list of patients’ appointment record by calculating sampling interval as K=N/n (where N (125) average number of patients per day; n (18) is sample to be taken per day). Then participant’s medical card number (ID) were taken every seventh interval for comprehensive chart review. A colored sticker was posted on the patient chart to identify easily during follow up and to avoid double recruitments.*

3. Please include a copy of the original language version of the survey or questionnaire used in the study to ensure that you have provided sufficient details that others could replicate the analyses. Please include a copy, in both the original language and English, as Supporting Information.

- *A copy of the original language(Amharic) and English version of the survey or questionnaire used in the study has been provided as supporting information.*

**Response to Reviewers**

**Reviewer #1**

**Abstract**

- One the abbreviation MTM is introduced, it should be used throughout the abstract.
- *Accepted and corrected*
- The methods should describe who delivered the intervention (pharmacists):
- *The interventions were provided by pharmacist in collaboration with physicians and nurses*.
- Methods should also describe inclusion criteria – was the MTM clinic for all people with DM, or just Type 2? What about gestational diabetes? In short – define the type(s) of diabetes patients included
- *The study included all adult patients who had been diagnosed with diabetes (both type I&II) and had been taking anti-diabetes medications for at least three months. Patients with gestational diabetes, those who decided to change their follow-up clinic, and those who refused to participate in the study were excluded.*

**Introduction**

The introduction would benefit from making distinctions between types of diabetes – epidemiology and management differs by type and this should be reflected in the opening 2 paragraphs.

*….. Based on ethiopathogenesis of the disease diabetes can be classified as type I diabetes(5 to 10%), type II diabetes(∼90–95% of diabetes), gestational diabetes mellitus (GDM) and monogenic diabetes and secondary diabetes(less common) (3). The management of diabetes depend on understanding of the pathophysiology of the disease. In type I diabetes mellitus, there is a significant insulin deficiency and the only therapeutic option is the administration of insulin or insulin analog. Metformin in combination with insulin may help type I DM patients who are overweight, taking high doses of insulin, or have a HbA1c of more than 8%, according to recent research (3, 4).*

*While in type II diabetes mellitus individuals, have relative insulin deficiency and peripheral insulin resistance that require either oral medication or insulin/insulin analogue or both. The currently available class of oral antidiabetes medication include sulphonylurea(SUs) and metiglinides, which function as insulin secretagogues and promote insulin secretion directly; biguanides (such as metformin) and thiazolidinediones (TZD), which improve insulin sensitivity; and alpha glucosidase inhibitors, which minimize the need for post-prandial insulin secretion by slowing intestinal carbohydrate absorption (2). …*

*2. International Diabetes Federation. IDF Diabetes Atlas. 8th ed. Brussels, Belgium: International Diabetes Federation. (2017). Available at: http://www. idf.org/diabetesatlas. Accessed Feb 15, 2020.*

*3. World Health Organization (WHO). Global Report on Diabetes. 2016.* *Available at:https://apps.who.int/iris/bitstream/handle/10665/204871/9789241565257_eng.pdf;jsessionid=4078DF16BC4D5264F70EE490988E4FBB?sequence=1 Accessed Feb 15, 2020.*

*4. Meyer L, Guerci B. Metformin and insulin in type 1 diabetes: the first step. Diabetes Care. 2003;26(5):1655-6.10.2337/diacare.26.5.1655-a*

Methods

Again, the authors need to describe whether the study population included type 1 and type 2 patients. It is here we learn that those with gestational diabetes were excluded.

- *All adult patients diagnosed with DM (both type I&II) and on anti-diabetes drugs for at least three months were included in the study.*

Page. 9 HgA1c should be HbA1c – also on page 12, 19, 23

- *The term HgA1c mistakenly written in the manuscript in some area were corrected(HbA1c)*

Results
In table 1 we finally learn that both type 1 and type 2 patients are included in this study. This is too late – it should be clear from the abstract and the introduction needs to address the differences in type 1 and type 2 epidemiology and management.

- *The comment is addressed*

In table 2, the column containing ‘paired differences – mean’ is inconsistent. The change in mean appears to negative for every variable, yet only two (WC, DBP) of them are presented as negative values. I suggest this table is checked carefully for consistency

- *Descriptive Statistics: In this expression, the mean was calculated directly from the descriptive analysis using the continuous variables.*
- *Paired Differences: Paired sample t-test was used to ascertain the level significance of the differences between the baseline mean during the initiation of the service and end of MTM follow up. In this analysis, some outlier variables might be removed.*
- *Because of this, the mean obtained from descriptive analysis and paired sample t-test could have litter difference. In table 2, the first mean is the mean obtained from descriptive analysis while the paired difference the mean difference obtained from paired t-test analysis.*
- *The purpose of the test is to determine whether there is statistical evidence that the mean difference between paired observations is significantly different from zero.*

**Discussion**

I would encourage the authors to be more circumspect in their language about causation e.g. “The reduction is obviously ascribed to the intervention, which provided targeted optimization of medication therapy need, lifestyle modification and enhanced medication adherence through consultations.” – as this is not a randomized controlled trial (RCT), I would use more tentative language – e.g. ‘our findings strongly suggest that the intervention reduces HbA1c’. This applies to all of the reductions/positive changes that the study reports on.

- *The comment is accepted and implemented*

*….. The reduction is strongly attributed to the intervention, which provided targeted optimization of medication therapy need, lifestyle modification and enhanced medication adherence through consultations….*

The authors should note the need for an RCT to confirm findings in the limitations section.

- *The comment is accepted and implemented*

*… To confirm the current finding and reduce the bias conducting an RCT is important….*

**Reviewer #2**

1. The title, Patient outcomes and satisfaction improves following introduction of Medication therapy management service at the ambulatory diabetes clinic of Tikur Anbessa Specialized Hospital, Addis Ababa, Ethiopia”, is a biased one from the onset. The word improves shows bias. The title should rather be, “Impact of Medication therapy management service on Patient outcomes and satisfaction in the ambulatory diabetes clinic of Tikur Anbessa Specialized Hospital, Addis Ababa, Ethiopia”

2. Again, satisfaction is an outcome (humanistic outcome), so it is like a repetition. The authors could modify the title further to ‘sellected clinical and humanistic outcomes’

3. The final title should now be,”Impact of Medication Therapy Management service on sellected Clinical and Humanistic outcomes in the ambulatory diabetes Patients of a Specialist Hospital in Addis Ababa, Ethiopia” or ,”Impact of Medication Therapy Management service on sellected outcomes in the ambulatory diabetes Patients of a Specialist Hospital in Ethiopia”

- *Accepted Title:* ***“Impact of Medication Therapy Management service on selected Clinical and Humanistic outcomes in the ambulatory diabetes Patients of Tikur Anbessa Specialist Hospital, Addis Ababa, Ethiopia”***

4. The first sentence at the background section of the abstract is rather long and passive. It should be split into two and the phrase, ‘as they often’ should be exponged. Line 17

- *Corrected as “Diabetes mellitus (DM) patients are at increased risk of developing drug therapy problems (DTPs). The patients had a variety of comorbidities and complications, and they were given multiple medications.”*

5. The third sentence of the background section; (Thus aim of present study was to assess .....) could be rephrased to, (The study assessed .......) Line 19

- *“The study assessed the impact of provision of MTM service on selected clinical and humanistic outcomes of patients at the diabetes mellitus clinic of Tikur Anbessa Specialized Hospital (TASH).”*

6. In lines 19- 21, the objective of the study was incomplete eg the impact of MTM on what? Patient outcomes, satisfaction, etc

- *“The study assessed the impact of provision of MTM service on selected clinical and humanistic outcomes of patients at the diabetes mellitus clinic of Tikur Anbessa Specialized Hospital (TASH).”*

7. The outcome measures should be clearly delineated because patient outcomes comprises clinical, economic, and humanistic outcomes. Here, I guess the authors are referring to clinical outcomes; they should be specific on the them for precision.

- *Corrected as “Clinical and humanistic outcomes”*

8. Line 66 should mark the beginning of a new paragraph; (It also involves .....) should translate to (Also, it involves .....)

*Corrected as… Also it involves providing self-management education…*

9. The phrase in line 103 (so as double recruitment ....) should be rephrased

- *A colored sticker was posted on patients charts to avoid double recruitment.*

10. In line 133, indicate the major modifications made to the questionnaire and reference it.

*The Strand classification was initially created to help pharmacists’ engagement in ensuring the best possible drug treatment and patient safety.* *Pharmacists use the classification to establish standards of practice. Drug related problem (DRP) was described by Strand and colleagues as "an unfavorable patient experience involving drug therapy that actually or potentially interferes with a desired patient outcome." According to the Strand and colleagues,* e*ight categories of DRPs were identified (Strand et al. 1990).* *This classification did not distinguish between problems and causes.* *Later, the classification was revised by renaming DRPs as DTPs (Drug-Therapy Problems), also drug interactions were removed from problem category to cause category as they were considered as causes for DTPs (Cipolle et al. 1998, Cipolle et al. 2004). Thus, the revised classification included 7 problem categories and 33 cause categories. The latest revision of the classification includes 7 problem categories and 37 cause categories (Cipolle et al. 2012).*

- *Strand LM, Morley PC, Cipolle RJ, Ramsey R, Lamsam GD. Drug-related problems: their structure and function. DICP 1990;24:1093–1097.*
- *Cipolle R, Strand L, Morley P. Pharmaceutical Care Practice. The Clinicianʼs Guide. McGraw-Hill Medical Publishing Division, New York. 1998.*
- *Cipolle R, Strand L, Morley P. Pharmaceutical Care Practice. The Clinicianʼs Guide. 2nd edition. McGrawHill Medical Publishing Division, New York. 2004.*
- *Cipolle R, Strand L, Morley P. Pharmaceutical Care Practice: The Patient Centered Approach to Medication Management Services. 3rd edition. McGraw-Hill Medical Publishing Division, New York. 2012.*

11. How was bias handled/eliminated from the study?

*Bias handling*

*Bias is an avoidable in scientific research. It may happen at any point during the research process, including during the study design, data collection, study implementation, data analysis, and so on. In current study, we try to minimize the bias in the study in the following methods.*

*1. Selection bias:*

- *Uniform data collection tool was used throughout the study period, which can minimize inter-observer variability during gathering and entering data.*
- *A systematic random sampling technique was used based on list of patients’ appointment record by calculating sampling interval. Only patient card number was considered for initial selection.*
- *Participants patients originate from the same general population.*
- *The study design is prospective which may avoid selection bias as outcome is unknown at time of enrollment.*
- *The sample size was enough to conclude to the study population from the data analysis. Large sample size also reduce the confounding.*
- *There is clear inclusion and exclusion criteria.*

*2. Bias during study design*

- *The study design is pre-post prospective study which reduce the bias.*

*3. Data collection bias*

- *Data collection methods include questionnaires, laboratory, or medical chart review which was collected using pre-tested uniform data collection tool that minimize inter-person variability.*
- *Investigation result were obtained from chart and this measurement is uniform for all patients.*
- *Training was provided for data collector*
- *Pre-test was done on 5% of the sample at the diabetes clinic of TASH for completeness of variables before the baseline data collection started.*

*4. Data analysis*

- *The patient data is coded.*
- *Incomplete data were cleared before entering to software by principal investigator.*
- *The data analysis was conducted under supervision of biostatistician.*

12. The authors should clearly state the outcome measures of this study under a sub-title at the methods section of the manuscript

*Outcome Measures*

*… The main outcome measured were the change in DTPs, clinical and humanistic (treatment satisfaction) outcomes from pre-MTM(baseline) to post-MTM after intervention. The clinical outcomes include glycemic control (HbA1c, FBS, RBS), blood pressure, lipids profile (HDL, LDL, triglycerides) levels. DTP were determined by modified Cipolle(7) while the humanistic outcome treatment outcome were measured by satisfaction with medicines questionnaire (SATMED-Q)(25, 26). ……*

*7. Cipolle RJ, Strand LM, Morley PC. Pharmaceutical care practice: the patient-centered approach to medication management: 3rd ed McGraw Hill Professional (2012).*

*25. Rejas J, Ruiz MA, Pardo A, Soto J. Minimally important difference of the Treatment Satisfaction with Medicines Questionnaire (SATMED-Q). BMC Med Res Methodol. (2011) 11(1):142. doi: 10.1186/1471-2288-11-142.*

*26. Ruiz MA, Pardo A, Rejas J, Soto J, Villasante F, Aranguren JL. Development and Validation of the “Treatment Satisfaction with Medicines Questionnaire”(SATMED‐Q)©. Value Health. (2008) 11(5): 913-26. doi: 10.1111/j.1524-4733.2008.00323.x.*

13. In line 106, At the day of appointment, should be (On the day of appointment,)

*Corrected*

*…… on the day of appointment……*

14. The tables titles were rather to long. The authors should rephrase/restructure the tables titles to be very precise/concise; the name and location of the hospital can be deleted since it is already known from the title of the study.

*Corrected as*

- **Table 1:** Socio-demographic and clinical characteristics of adult patients
- **Table 2:** Clinical measures for mean scores and paired samples t-test for clinical outcome measures among adult diabetic patients
- **Table 3:** Drug therapy problems and causes among adult patients with diabetes
- **Table 4** Bivariate and multivariate analysis of factors associated with drug therapy problems among adult patients with diabetes
- **Table 5:** Relationship between treatment satisfaction and different characteristics of patients with diabetes

15. Line 246-251 need additional references to establish the statements made.

*Added reference*

*30.	Al-Azzam SI, Alzoubi KH, AbuRuz S, Alefan Q. Drug-related problems in a sample of outpatients with chronic diseases: a cross-sectional study from Jordan. Therap clinic risk manag. (2016) 12:233-9*

*31.	Redekop WK, Koopmanschap MA, Stolk RP, Rutten GE, Wolffenbuttel BH, Niessen LW. Health-related quality of life and treatment satisfaction in Dutch patients with type 2 diabetes. Diabetes care. (2002) 25(3):458-63*

16. In line 153, the statement (About 73% had) is undefined. The authored should define this population and be definite. Eg 75% of patients, type 2 diabetic patients, ???

*Corrected as*

*……In this study co-morbidities and complications were common among study participants, probably attributed to higher mean age (52 years) and duration of illness (14 years) as well as type of diabetes they had(most had Type 2 DM). …….*

17. Lines 271-272 should be elaborated with relevant studies/references

*Corrected as*

*This reduction was brought about by increased awareness of patients about lifestyle modification, side effect prevention and management, medication misuse, and importance of medication adherence (41, 42). In addition, it might be also due to a good communication created between pharmacist and physician during intervention in preventing the occurrence of DTPs (35, 43).*

*This reduction was brought about by increased awareness of patients about lifestyle modification, side effect prevention and management, medication misuse, and importance of medication adherence(43, 44). In addition, it might be also due to a good communication created between pharmacist and physician during intervention in preventing the occurrence of DTPs(37, 45).*

*37. Davis CS, Ross LAR, Bloodworth LS. The impact of clinical pharmacist integration on a collaborative interdisciplinary diabetes management team. J Pharm Pract. (2017) 30(3): 286-290. doi: 10.1177/0897190016631894.*

*43. Brummel AR, Soliman AM, Carlson AM, de Oliveira DR. Optimal diabetes care outcomes following face-to-face medication therapy management services. Popun health managt. (2013) 16(1): 28-34*

*44. Bukhsh A, Nawaz MS, Ahmed HS, Khan TM. A randomized controlled study to evaluate the effect of pharmacist-led educational intervention on glycemic control, self-care activities and disease knowledge among type 2 diabetes patients: A consort compliant study protocol. Medicine. (2018) 97(12):1-6*

*45. Strand LM, Cipolle RJ, Morley PC, Frakes MJ. The impact of pharmaceutical care practice on the practitioner and the patient in the ambulatory practice setting: twenty-five years of experience. Curr pharm design. (2004) 10(31):3987-4001*

18. In line 319 (The intervention period is relatively short) should be corrected

*Corrected as…. The intervention period is relatively short (six months) which may overestimate the outcome. The study is a single center study that might not allow to make generalization to the whole population…*

19. Line 298- 303, 319- 324 were poorly discussed and without any reference. The authors should improve on them.

*Corrected as…*

*…… The reduction is strongly attributed to the intervention, which targeted the optimization of medication therapy need, lifestyle modification and enhanced medication adherence through consultations. The awareness created through brochure, face-face and phone based education about the disease condition and its management; ADR prevention and management could also enhance treatment outcome(36-38)..…..*

*36. McAdam-Marx C, Dahal A, Jennings B, Singhal M, Gunning K. The effect of a diabetes collaborative care management program on clinical and economic outcomes in patients with type 2 diabetes. J Manag Care Pharm. (2015) 21(6):452-68. doi:10.18553/jmcp.2015.21.6.452*

*37. Davis CS, Ross LAR, Bloodworth LS. The impact of clinical pharmacist integration on a collaborative interdisciplinary diabetes management team. J Pharm Pract. (2017) 30(3): 286-290. doi: 10.1177/0897190016631894.*

*38. Boban B, Aswathy K, Athira B, Karthikeyan M, Xavier A, Aravind R. Impact of medication therapy management on knowledge, attitude and practice among diabetic patients. Clinic Epidem Global Health. (2017) 5(2):70-2*

20. The study was poorly concluded in lines 325-329.

Corrected as…

*…* *The study suggest that the provision of MTM service to patients with diabetes, with/without complications and comorbidity had the potential to improve the clinical parameters such as HgA1c, FBS and BP. The study also demonstrate that pharmacist led MTM service reduced the DTPs identified among ambulatory patients with diabetes in TASH. Recruiting patients to MTM service also increased treatment satisfaction among study participants. ……*

21. Page number is missing in reference NO 26, line 439. The first year (2011) on that reference should be cross checked.

*Corrected as……26(33). Lochner KA, Goodman RA, Posner S, Parekh A. Multiple chronic conditions among Medicare beneficiaries: state-level variations in prevalence, utilization, and cost, 2011. Medicare Medicaid Res Rev. (2013) 3(3) E1-E19. doi: 10.5600/mmrr.003.03.b02. ……*

22. The journal name in reference NO 28, line 446 should be cross checked.

*Corrected as …..28(35). Ndefo UA, Moultry AM, Davis PN, Askew R. Provision of medication therapy management by pharmacists to patients with type-2 diabetes mellitus in a federally qualified health center. Pharm Therap. (2017) 42(10):632-37 …*

23. Page number is missing in reference NO 36, line 473, and NO 37 line 475 respectively.

*Corrected as ….*

*36(48).Van Mourik MS, Cameron A, Ewen M, Laing RO. Availability, price and affordability of cardiovascular medicines: a comparison across 36 countries using WHO/HAI data. BMC Cardiovasc Disord. (2010) 10(25) 1-9. doi: 10.1186/1471-2261-10-25.*

*37(49).Zazuli Z, Rohaya A, Adnyana IK. Drug-Related Problems in Type 2 Diabetic Patients with Hypertension: A prospective Study. J Basic Clini Pharm. (2017) 8(4): 251-254*

**Reviewer #3**

**Abstract**

- The objective sentence beginning with ‘Thus aim of present study was to assess the impact’ should be revised by including the definitive article (the) where appropriate and using the case (or acronyms) for the terms medication therapy management and diabetes mellitus
- *The objective of the study is revised as “The study assessed the impact of provision of MTM service on selected clinical and humanistic outcomes of diabetes patients at the diabetes mellitus clinic of Tikur Anbessa Specialized Hospital (TASH).”*
- The sentence beginning with ‘Descriptive statistics and t-test, logistic regressions…’ should be revised
- *The sentence is revised as... Descriptive statistics, t-test, and logistic regressions were performed for data analyses. …*

**Introduction**

- The sentence ‘Drug-related hospital 60 admissions have also been significantly increased overtime’ should be revised. The word ‘been’ in the sentence is unnecessary
- *The statement is corrected as …* *Drug-related hospital admissions are also significantly increasing overtime.*
- The sentence ‘It account for 5-10% of admissions 61 and more than 50% of them were avoidable’ should be revised to ‘…and more than 50% of which are avoidable…’
- *The statement is corrected as:* *It account for 5-10% of admissions and more than 50% of which were avoidable.*
- The acronym ‘HgA1c’ is not commonly used. Consider using ‘HbA1c’ consistently throughout the manuscript.
  - *The term HgA1c mistakenly written in the manuscript in some area were corrected as HbA1c.*

**Methods**

**Design and patients**

- The last paragraph talks of 6000 adult patients annually with an average of 250 patients per week. Based on the estimates it seems like the setting sees around 13000 adult patients annually. Kindly clarify.
- The maximum appointment interval for follow-up at the DM clinic of TASH is six months for each patient. Depending on the patient's clinical condition (Glycemic control, presence of complication, and comorbidity) as well as patient load in the clinic, one patient can visit the clinic at least two times per year. As a result, approximately 6000 adult patients visit the clinic within six months; this ensures that the same patient number will attend the clinic for the next six months. As a result, around 6000 adult patients visit the clinic on an annual basis.
- Define the minimum age used to identify and include adult patients
- *Age more than 18years were considered for inclusion in the study.*

**Sampling**

- The first sentence talks of single proportion formula assuming a prevalence of 50%. Kindly clarify what measure was being used (i.e. prevalence of DTPs vs prevalence of medication nonadherence, etc.).
- *The prevalence of DTPs was used for calculation of sample size.*

**Intervention**

- In the first sentence, clarify what additional information was collected during the interviews with the recruited patients.
- *Additional information collected during the interviews from the recruited patients include* *social habit, economic status, educational and marital status, physical activity, etc.*
- In the assessment of DTPs, please clarify/justify why medication adherence not considered.
- Medication adherence was one of the outcome variables assessed during the MTM service provision. Due to the large volume of data, it was not included in the current manuscript. Separately, the manuscript's adherence portion is being prepared.

**Data collection and management**

- The sentence ‘However, the seventh category that assesses medication adherence was removed, as it to be assessed by MMAS tool…’ should be revised. It seems that the word ‘has’ is missing in the sentence. Also, the acronym MMAS should be defined in full and the reasons why medication adherence could not be assessed yet it was one of the outcome measures as indicated in the intervention section clarified.
- *The statement is corrected as……However, the seventh category that assesses medication adherence was removed, as it has to be assessed by Morisky medication adherence scale (MMAS) tool.*
- Medication adherence was one of the outcome variables assessed during the MTM service provision. Due to the large volume of data, it was not included in the current manuscript. Separately, the manuscript's adherence portion is being prepared.
- In the sentence starting with ‘Demographic (age, sex…) and clinical information (disease type…’, kindly clarify what lifestyle information was collected.
- *Lifestyle information includes physical activity, alcohol use, smoking and dietary status of study participants.*
- In the sentence starting with ‘Online resources (Micromedex, Launch Lexi-Interact’ kindly clarify and cite which standard textbooks were used for DTP identification.
- *Online tools (Micromedex, Launch Lexi-Interact^TM^) are used to identify and check for potential and actual drug interactions during DTP identification and intervention.*
- *The standard textbooks used for DTP identification were Applied therapeutics : the clinical use of drugs 11^th^ ed and Pharmacotherapy A Pathophysiologic Approach, 10e*.

*28. Zeind CS, Carvalho MG. Applied therapeutics : the clinical use of drugs (2018) Available from: http://pharmacy.lwwhealthlibrary.com/book.aspx?bookid=2324.*

*29. DiPiro JT, Talbert RL, Yee GC, Matzke GR, Wells BG, Posey L. Pharmacotherapy A Pathophysiologic Approach, 10e. New York, NY: McGraw-Hill Education LLC; (2017)*

- In the sentence starting with ‘The role of the pharmacists was identifying DRPs and providing intervention package …’ the acronym DRP is used but was not introduced before. Consider using DTP consistently throughout the manuscript.
- *The comment is accepted*

**Data Analysis**

- In the sentence ‘Descriptive analysis was computed as frequency, percent, mean and standard deviation (SD) for continuous and categorical variable’ clarify which specific statistics were computed for continuous vs categorical variables (e.g., means vs proportions).
- Descriptive analysis was computed as frequency and percent for categorical variable’ and mean and standard deviation (SD) for continuous variables.
- Clarify the regression analysis model used to assess DTPs, the variables that were in the model, and the outcome that was evaluated.
- To examine the influences of different variables on DTPs logistic regression analysis model were used. Demographic and *clinical information of the patient were the variable used in the model while DTPs(Yes/No) were the outcome variable that was evaluated.*

**Results**

**Socio-demographic and clinical characteristics of patients**

- Kindly clarify whether Table 1 refers to the baseline characteristics of the study population i.e before the intervention
- *Table 1 shows the baseline characteristics of the participants recruited for the intervention.*
- Kindly clarify the criteria used to define hyperglycemia
- *Hyperglycemia was defined as an average FBG level of above 130 mg/dl for patients between 18 and 60 years old with no comorbid with disease duration of below 8years and values above 150 mg/dl for those above 60 years of age and patients with multiple comorbid and also those with disease duration more than 8 years (2, 24, 27).*

*2. International Diabetes Federation. IDF Diabetes Atlas. 8th ed. Brussels, Belgium: International Diabetes Federation. (2017). Available at: http://www. idf.org/diabetesatlas. Accessed Feb 15, 2020*

*24.* *American Diabetes Association. Standards of Medical Care in Diabetes-2018. Abridged for Primary Care Providers. Diabetes Care. (2018). Available at: https://clinical.diabetesjournals.org/content/41/ Supplement_1. Accessed April 14, 2020*

*27. The Society for Endocrinology, Metabolism and Diabetes of South Africa Type 2 Diabetes Guidelines Expert Committee. SEMDSA 2017 guidelines for the management of type 2 diabetes mellitus. J Endocr Metab Diabetes S Afr. (2017) 22(1 Suppl 1):S1-S196*

- Kindly clarify the sentence starting with ‘About 73% (299) and 37% (151) had…’
- *Comorbidity and complication were found in 73 % (299) and 37 % (151) of the study participants, respectively. Hypertension (56.2%) and neuropathy (30.1%) were the two most common comorbidities and complications, respectively (Table 1).*

***Drug therapy problems***

- In the last sentence in the first paragraph, the acronyms ACEI and ARBs should be defined in full the first time they occur in the text.
- *Corrected as …… and angiotensin converting enzyme inhibitors (ACEI)/ angiotensin-receptor blockers(ARBs) (49.8%)……*
- The analyses conducted seem to include a number of individual hypotheses tested simultaneously. This could result in a multiple hypothesis testing problem that the authors should address when describing results that were found significant.
- *In current study each outcomes was assessed separately. This individual analysis reduce the problem of multiple hypothesis testing that occurred when there are multiple outcomes of interest and it is desired to determine on which of these outcomes a treatment has an effect.*

**Treatment Satisfaction**

- Define the range of the treatment satisfaction measure
- *The satisfaction rate ranged from 88.3 (medical care domain) to 91.6 (convenience domain).*

**Discussion**

- Several results that had previously been described in the results section seem to be repeated in the discussion. This is redundant and affected sentences should be revised accordingly.
- *The comment is accepted and correction is done on the manuscript.*

**Clinical outcomes**

- The title of the subsection should read ‘Clinical Outcomes’
- *Corrected*
- The clinical relevance of the reduction in HbA1c compared to the baseline should be clarified. What amount of reduction in HbA1c would be considered clinically significant for the purposes of the management of the diabetes mellitus?
- *In present study MTM intervention reduced HgA1c in about 0.92%. Improvement in glycemic control has been shown to result in fewer microvascular and slowed progression of macrovascular complications(Nadine Shehab, 2017). In study of Stratton et al., (2000) showed that a 1% decrease in serum HbA1c corresponds to a significant decreased risk of complications. In that study this amount reduction of HgA1c also cause 43% reduction in amputation or fatal peripheral blood vessel disease, 37% reduction in microvascular complications eg kidney disease and blindness, 21% reduction in all deaths, 14% reduction in heart attacks and 12% reduction in strokes(Stratton et al., 2000).*

*Nadine Shehab M.C.L., Andrew I. Geller, Kathleen O. Rose, Nina J. Weidle, and Daniel S. Budnitz, 2017. US Emergency Department Visits for Outpatient Adverse Drug Events, 2013-2014. JAMA, 316, 2115–2125.*

*Stratton I.M., Adler A.I., Neil H.A.W., Matthews D.R., Manley S.E., Cull C.A., et al. 2000. Association of glycaemia with macrovascular and microvascular complications of type 2 diabetes (UKPDS 35): prospective observational study. Bmj, 321, 405-412.*

- The sentence beginning with ‘The reduction is obviously ascribed to the intervention’ is potentially misleading due to the term ‘obviously’. It should be revised in terms of likelihood and uncertainty of the associations/correlations measured.
- Corrected as…..*The reduction is strongly attributed to the intervention, which provided targeted optimization of medication therapy need, lifestyle modification and enhanced medication adherence through consultations…..*

**Drug therapy problems**

- The explanation of why males were more likely to develop DTPs post-intervention should be cited or clarified as the authors' interpretation.
- *The male gender (AOR=3.06, 95% CI: 1.54-6.07) was more likely to develop DTPs than the female gender. This was in line with previous studies conducted in Malaysia (10) and Jordan (37). The likely explanation could be related to differences in health behavior as well as acceptance of recommendation between males and females(50, 51).*

*[50]	Campbell AN, Nunes EV, Pavlicova M, Hatch-Maillette M, Hu M-C, Bailey GL, et al. Gender-based outcomes and acceptability of a computer-assisted psychosocial intervention for substance use disorders. Journal of substance abuse treatment. 2015;53:9-15*

*[51]	Schofield T, Connell RW, Walker L, Wood JF, Butland DL. Understanding men's health and illness: a gender-relations approach to policy, research, and practice. Journal of American college health. 2000;48(6):247-56*
